# Bibliography

**Published:** 1899-04

**Authors:** 


### Bibliography.

Three Thousand Questions on Medical Subjects arranged for Self-Examination. Second Edition. Enlarged. P. Blakiston, Son & Co., Philadelphia, 1899.

This small book, suitable for carrying in the pocket, has a certain value for medical students, obliged to keep constantly in mind a great multitude of facts. The answers to questions are here wisely omitted. This means, necessarily, research and a positive impression upon the mind. The questions cover reasonably well the various subjects. The questions on dental pathology are by no means satisfactory, and should, in future editions, be added to or omitted altogether.

TRANSACTIONS OF THE IOWA STATE DENTAL SOCIETY AT THE THIRTY-FIFTH ANNUAL MEETING HELD AT DES MOINES, May 3, 4, 5, and 6, 1898.

There is always a satisfaction, in looking over the transactions of a society, in having them placed before the reader in clear type and tasteful binding, and this Society is to be congratulated that its proceedings are presented in a manner that invites a careful examination of the contents.

There is much in this volume worthy of preservation, but it would have been better had the blue pencil of the editor been more freely used. It is a mistake, too often made, to publish everything reported by the stenographer. The crudities given out in discussion are the terror of all editors, but while they suffer by virtue of their office, the lapses from good English should not be permitted to go out to the world in cold type. These can only be eliminated by erasing without fear or favor.

